# Isolation, Identification, Whole-Genome Sequencing, and Annotation of Four *Bacillus* Species, *B. anthracis* RIT375, *B. circulans* RIT379, *B. altitudinis* RIT380, and *B. megaterium* RIT381, from Internal Stem Tissue of the Insulin Plant *Costus igneus*

**DOI:** 10.1128/genomeA.00847-15

**Published:** 2015-07-30

**Authors:** Steven J. Polter, Alexander A. Caraballo, Yin P. Lee, Wilhelm W. H. Eng, Han M. Gan, Matthew S. Wheatley, Michael A. Savka, Bolaji N. Thomas, André O. Hudson

**Affiliations:** a Thomas H. Gosnell School of Life Sciences, Rochester Institute of Technology, Rochester, New York, USA; b College of Health Science and Technology, Rochester Institute of Technology, Rochester, New York, USA; c Monash University Malaysia Genomics Facility, Monash University Malaysia, Selangor, Malaysia; d School of Science, Monash University Malaysia, Selangor, Malaysia

## Abstract

Here, we report the isolation, identification, whole-genome sequencing, and annotation of four *Bacillus* species from internal stem tissue of the insulin plant *Costus igneus*, grown in Puerto Rico. The plant is of medicinal importance, as extracts from its leaves have been shown to lower blood sugar levels of hyperglycemic rats.

## GENOME ANNOUNCEMENT

The pantropical plant *Costus igneus*, commonly referred to as the insulin plant, belongs to the *Costaceae* family and is considered a medicinal plant due to its antidiabetic properties. Recent studies have shown that extracts from the leaves of *C. igneus* were able to lower the blood sugar levels of hyperglycemic rats (1). In addition to its medicinal properties, phytochemical screenings of extracts from *C. igneus* revealed that iron, ascorbic acid, α-tocopherol, and β-carotene are in high abundance (2). We were interested in the isolation and identification of endophytic bacteria that associate with *C. igneus*, to gain some insights into the plant-bacteria symbiotic relationships. This study was facilitated by isolation, initial identification, and whole-genome sequencing and annotation of bacteria that associate with the plant. Briefly, internal tissue from a surface-sterilized stem, obtained from a farm in Puerto Rico, was used to inoculate tryptic soy broth (TSB) medium. Cultures were then serially diluted and plated on several media (tryptic soy agar, Luria agar, nutrient agar, potato dextrose agar, and R2A), which subsequently led to the isolation of several bacteria. Four Gram-positive bacteria were chosen for further analysis based on colony morphology and microscopic examination following Gram staining. The bacteria were identified as *B. anthracis*, *B. circulans*, *B. altitudinis*, and *B. megaterium*, based on nucleotide sequence analysis of the variable 3 (V3) region from the 16S rRNA gene amplified using the PCR primers V3-forward (5′-ACTCCTACGGGAGGCAGCAG-3′) and V3-reverse (5′-ATTACCGCGGCTGCTGG-3) (3).

Genomic DNA was isolated from the endophytes using the GenElute bacterial genomic kit (Sigma-Aldrich, St. Louis, MO, USA) and prepared for whole-genome sequencing using the Nextera XT library preparation kit (Illumina, San Diego, CA, USA). Whole-genome sequencing was performed on the Illumina MiSeq (2 × 150-bp paired-end reads) located at the Monash University Malaysia Genomics Facility. The reads were error corrected and assembled *de novo* using SPAdes version 3.5 (4). Scaffolding of the contigs and *in silico* gap closing of the resulting scaffolds were performed using SSPACE and GapFiller, respectively (5, 6). Genome annotation was performed using the NCBI Prokaryotic Genomes Annotation Pipeline. The key attributes for each of the four genomes and annotations are summarized in Table 1.

**TABLE 1:** Sequencing and annotation details of the four *Bacillus* species isolated from *Costus igneus*

Strain	BioProject no.	BioSample no.	Accession no.	Organism	Genome coverage (×)	Genome size (bp)	No. of contigs	No. of ORFs^*a*^	No. of tRNAs	No. of rRNAs
RIT375	PRJNA285407	SAMN03753508	LDPG00000000	*Bacillus anthracis* RIT375	66	5,677,918	62	5,666	98	15
RIT379	PRJNA285407	SAMN03753509	LDPH00000000	*Bacillus circulans* RIT379	71	5,454,271	103	4,950	85	22
RIT380	PRJNA285407	SAMN03753582	LDPI00000000	*Bacillus altitudinis* RIT380	97	3,972,159	85	3,985	87	9
RIT381	PRJNA285407	SAMN03753583	LDPJ00000000	*Bacillus megaterium* RIT381	41	5,864,214	240	5,616	151	41

a ORFs, open reading frames.

A summary of the secondary metabolite analysis of the four species using the antibiotics Secondary Metabolite Analysis Shell (antiSMASH) tool revealed interesting results regarding the identification of gene clusters that are involved in the synthesis of secondary metabolites. For example, the genome of *Bacillus anthracis* RIT375 resulted in the identification of gene clusters for the synthesis of a lasso peptide in contig 1 and a petrobactin in contig 3, and *Bacillus circulans* RIT379 possess gene clusters for lantipeptide and cepacian in contigs 7 and 11. *Bacillus altitudinis* RIT380 possess gene clusters for bacilysin, lichenysin, and sporulation killing factor in contigs 8, 9, and 13, respectively. *Bacillus megaterium* RIT381 possessed gene clusters for surfactin and carotenoid in contigs 1 and 3 (7, 8).

### Nucleotide sequence accession numbers.

The nucleotide sequences have been deposited at DDBJ/EMBL/GenBank under the accession numbers listed in Table 1.
